# Some Observations on Tea-Poisoning
1Read at a Meeting of the Bristol Medico-Chirurgical Society, November 8th, 1911


**Published:** 1911-12

**Authors:** Newman Neild

**Affiliations:** Lecturer on Pharmacology and Therapeutics, University of Bristol; Physician to the Bristol General Hospital, with charge of Out-patients


### SOME OBSERVATIONS ON TEA-POISONING.[1]

BY

NEWMAN NEILD, M.B., M.R.C.P.,

*Lecturer on Pharmacology and Therapeutics, University of Bristol ;*
*Physician to the Bristol General Hospital, with charge of Out-patients.*

TEA-POISONING is more often a complication of previously-existing disease than it is the sole cause of the patient's complaint.

The commonest co-existing diseases are those which most frequently lead to excess in tea-drinking. First amongst these is oral sepsis, due to decaying stumps of teeth, septic gums and pyorrhœa alveolaris. During the night the undisturbed incubation of bacteria in the mouth and the accumulation of their products creates a bad taste, that is removed for a time by an early-morning cup of strong tea; the more tannin present in the infusion the more efficient for the purpose. With this early cup of tea we have the best conditions for the rapid absorption of a poison. The solution is hot and concentrated, the stomach has had a long rest, and it may be an hour or more before any food is taken. But the presence of a bad taste in the mouth is not the only cause of the craving for tea in these cases ; the absorbed

[1] Read at a Meeting of the Bristol Medico-Chirurgical Society, November 8th, 1911.

toxins from the septic trouble, both by their direct action upon the nervous system and by the starvation of that system by the secondary anæmia, produce a condition which urgently calls for the stimulant action of tea. The decomposition is also responsible for various disturbances of the digestion, and the tea which is taken to relieve the trouble may in turn produce further disturbances on its own account.

In women at the menopause nervous instability is a frequent cause of excess in tea-drinking; and although tea seems to brace the nerves for a short time, it also greatly aggravates certain of the symptoms.

Amongst the working-classes the woman who before marriage has lived an active life, and then settles down to the management of a house of her own, very frequently "takes to tea." Each day the household work occupies her for but a short time, after which she has a long, lonely and idle day, the dreariness of which is only relieved by the teapot. Hence the longer she remains childless the more liable is she to the habit.

Certain occupations give much opportunity for this excess, especially those of tailoress and domestic servant. In another sphere of existence the indefatigable afternoon-caller often succumbs to the perils of her calling.

In out-patient practice there are few more tragic figures than that of the old woman who evades the shame of the workhouse by the hopeless heroism of solitary starvation. Her aged body, shrivelled and discoloured, irresistibly recalls some lines from "Les Regrets de la Belle Heaulmière." Tea, her sole consolation, is at the same time the cause of much of her misery. "Thus fareth many and many a one."

Amongst men those whose occupations create thirst, with the marked exception of brewers' employés, are liable to become excessive tea-drinkers, although it is in sedentary occupations that the effects of tea excess are most rapidly produced. A very large amount of tea is often taken by those whose occupation makes them perspire freely, without producing any distressing symptoms.

The fact, noted by Beard, that coffee is less harmful in hot and in cold countries than in temperate ones may be explained in part by the greater activity of the skin in a hot climate and of the kidneys in a cold, with the result that elimination is more rapid than in temperate temperatures. With the onset of cold weather men perspire less freely at their work, and may show signs of poisoning if they take the same amount of tea as formerly. I have observed this connectionrin several cases.

It is noticeable that amongst working men it is the steady, intelligent, and thrifty man with a comparatively high standard of comfort who is most liable to suffer from this poisoning. In marked contrast to the alcoholic member of society, he frequently suffers from neurasthenia due to a wise limitation of the family by unwise means. The resulting neurasthenia calls for tea.

In a large number of my cases the question as to the form and the amount of the beverage indulged in was reserved until all the symptoms had been elicited ; but since certain questions were only asked where tea excess was suspected, it cannot be said that all bias was removed. It has been in comparatively rare cases that tea excess has been present as the sole causal factor, and even in some of these it is hard to deny that some of the symptomatology may be due to a sedentary life.

In the majority of cases of chronic tea-poisoning the symptoms come on very gradually during some months, and then rapidly increase during the last few weeks or days before the patient comes under observation.

What are the symptoms which should lead one to suspect tea-poisoning ?

A celebrated surgeon once asked his house-surgeon, for the purposes of a clinical lecture, to look through the case books and collect a few typical cases of a certain affection of the knee-joint. The case books for some years back were diligently searched, and a number of histories were selected, condensed, and forwarded to the surgeon. Not one proved typical. The house-surgeon was reproved and urged to make further efforts, with the result that several typical cases were discovered admirably adapted to the celebrated surgeon's requirements.

The lecture was a success, but there were three conscience-stricken residents when that clinical lecture appeared in print. But I am convinced that the most truly typical case is the constructed one, and venture to set forth the following :—

M. N., aged 26, married eighteen months, no children ; formerly in good health as a factory hand. About a year ago began to suffer from dizziness and headache ; felt very run down, and became irritable. Could assign no cause for her illness. Has no pecuniary or other troubles. Later became sleepless, or woke up in the night with attacks of difficult breathing. During the day easily became short of breath, and had frequent " fainty " attacks, and often suffered from " fluttering " of the heart or attacks when the heart " thumped." Noticed about this time that her former good complexion and colour had been supplanted by pallor and a yellowish tinge ; the last fortnight she has suddenly become a brownish tint. Suffers from pain in the chest, chiefly behind the lower part of the breast-bone ; has " wind " sometimes, but it has only been really troublesome during the last fortnight, now that constipation has been added to her troubles. About an hour and a half after meals there is a sinking sensation in the stomach. The sensation is relieved at once by tea or food. There is great muscular weakness, and particularly the legs seem to fail her at times. Tea now causes the face to flush, or even seems to cause flushing over the whole body at times. Hot broth or soup does not produce anything like so great an effect. Since the skin became dark there has been much irritation of the skin. The last few days the sight of food makes her feel sick, and she has now turned against tea, but still takes a little.

There is great and even to herself unaccountable depression : often sits down and has a good cry without deriving that benefit therefrom to which she was formerly accustomed. Is very nervous : a knock at the door, and the heart stops suddenly, and swells as if it would choke her ; then clammy cold, trembling and faint, she is overwhelmed by the idea that someone has come to bring a terrible calamity upon her. She hardly dares to open a letter lest it contain bad news, upbraiding, or accusations. Only the necessity of shopping prevents her from remaining indoors the whole time. For some months two cups of strong tea have been taken with each meal, as well as an early cup which her husband prepares and brings up to the invalid every morning, and an occasional cup when she feels so disposed. When friends drop in to cheer her up she pours out her woes and a few more cups of tea.

The patient looks tired and anxious. The hair is of medium brown, the eyes grey ; the skin is tinted a very pale brown, darker round the eyes, at the temples, the margin of the forehead, and below the mouth. The neck is very dark where the collar fits closely. The abdomen is somewhat darker than the thorax. On closing the eyes there is tremor of the lids, and a slight tremor of the hands when the fingers are widely spread and extended. The palms of the hands are moist. The tendon reflexes are increased. There are no decayed teeth or pyorrhœa alveolaris. The breath is not exactly disagreeable, although it has a strong and characteristic odour. The heart is nervous, increasing in rapidity for a few moments on the least excitement, and the area of cardiac dulness varies during examination like that of the chlorotic patient. The patient is aware of the irregularity, and describes the sensation when the beats become uncountable for a second or two as a "fluttering," or as if the "heart turned over," and there is a feeling of fulness in the chest, and she becomes conscious of some difficulty in breathing. The pulse is variable both in volume and rapidity. The blood-pressure is normal. The breathing is sometimes shallow, sometimes sighing with lengthened pauses. The blood shows no particular changes beyond some degree of anæmia. The urine is very pale, of low specific gravity, and contains no albumen or sugar.

The above case represents a severe and uncomplicated case of tea-poisoning.

To refer to some of the symptoms more fully.

Many of these symptoms are much more prominent and more rapidly produced where there is also sepsis in the mouth or constipation present, particularly the discolouration of the skin and the dyspepsia. At one time I thought that the constipation was the cause of the discolouration, but several "well-marked" cases have had no trouble with the bowels. Two of these cases were tested, one with carmine and the other with charcoal, to make certain that the regular motions were not delayed stools. Symptoms attributable to the colon are not uncommon, but that common cause of discolouration, a dilated colon, was found only in a small number of the cases where it was looked for. The roughening of the back of the upper arm, as described by Arbuthnot Lane in dilatation of the colon, is not unusually common in tea poisoning. The whites of the eyes are not discoloured, thus distinguishing the discolouration from a slight jaundice.

To digress into theory for a moment. The colour is probably produced by some toxin due to disturbances of digestion, caused in these cases by tea, and not to the direct action of any absorbed constituent of the tea, although doubtless the frequent flushing due to the tea greatly assists the action upon the skin. The pigment is not a deposit in the skin, but a product of the skin. A patient who told me that she could "drink tea until she looked like it" was nearer the truth than she realised. At the time when discolouration is making most rapid progress there is usually some irritation of the skin, and in some cases this irritation is severe. In cases of habitual constipation and oral sepsis the pigmentation comes on earlier in the case and is more pronounced. Tremor also is more common where there are other causes of neurasthenia at work.

The hot flushes so frequent in tea-poisoning are, as one would expect, much more distressing at the menopause. Those who suffer from acne rosacea, or eczema, are much more conscious of the flushing than others; but as to any causal relationship between tea and the acne rosacea it is difficult for me to express an opinion of any value, since in hospital practice all well-marked cases of acne rosacea are sent directly to the physician for diseases of the skin. But without any observations being particularly directed to the point, this skin disease was noted as present in five out of the last 109 cases which I have had under my care. Certainly tea adds considerably to the distress of these patients, and one woman tells me that whenever she drinks a cup of tea "all the blotches on me face throb as if you had a gathering." Symptoms of gout are not noticeably more frequent amongst those suffering from tea excess, although tea delays recovery and increases the trouble in gouty attacks.

Occasionally a patient will complain of polyuria. One case that came under my care had actually been admitted into a medical institution in Bristol for "drinking diabetes." She had rapidly improved under treatment, but on leaving the institution the trouble became as bad as ever. The absolute prohibition of tea promptly stopped the polyuria. Here a few experiments proved the relationship of tea to the complaint.

There is only one pathognomonic sign of tea excess, but it is not present in all cases; that is, the odour of the breath. It is difficult to describe a smell, but the nearest smell to which I can compare it is that of musty old books. Without being unpleasant, it is often so strong as to be detectable even where there is also a fœtid smell of oral sepsis. It is secreted by the lungs, and at times provides a clue to the cause of the patient's trouble. Some teas cause the smell far more than others do. Similarly, in the case of another beverage, the so-called "peaty" quality, of which certain brands boast, will often provide through the patient's breath a valuable hint of excess long before the smell of alcohol or acetone can be detected. Here also it is excreted by the lungs.

The cardiac symptoms are more akin to those of tobacco-poisoning than to those of alcoholism, the symptoms of nervous derangement predominating over the signs of muscular degeneration. From our knowledge of the action of caffein upon the respiratory centre, we might presume that the respiratory disturbance was due to reaction from, or over-action of, this direct stimulation of the centre; but from a clinical point of view we cannot entirely ignore the disturbance of the heart or the dyspepsia as causal factors. In six cases of tea-poisoning I have found that the blood-pressure is not much altered, although in one very severe case it was lowered. Arterio-sclerosis is not more common in these cases than would be accounted for by its general frequency, yet it must be admitted that the three types of the preliminary stage of arterio-sclerosis, as set forth by Stengel, would cover the great majority, if not all, of the cases of chronic tea-poisoning. His description of the "nutritional," "neurasthenic" and "nervous" types must include in its embrace some toxæmias that do not proceed to arterio-sclerosis. Some disturbance of the digestion is always present. Myasthenic or atonic dyspepsia may serve as a general hold-all for the symptoms of the indigestion from which these cases suffer, yet there are one or two symptoms which require especial reference.

A frequent symptom is a peculiar "sinking sensation," that comes on with striking regularity about an hour and a half after having taken tea, whether with or without food. It comes on quite suddenly, and begins with a feeling of movement or contraction at the pyloric end of the stomach. It is caused by excess of tea, and yet this excess may consist of the only cup taken in the twenty-four hours, provided that the tea and its method of preparation are sufficiently bad. Personal experience first drew my attention to the symptom, and it has been noted by other observers.

Constipation is by no means always present, but is usual, and when it does occur greatly hastens the onset of the severe symptoms. As is the case with other astringents, tea may sometimes cause diarrhœa. In one case under my care the neurasthenic symptoms, combined with the alternation of constipation and diarrhœa, led me to diagnose mucous colitis, yet the intestinal symptoms ceased when the tea was withheld. Many patients complain of regurgitations, and these are more often described as "burning" or "bilious" than acid. The loss of appetite is partially responsible for the wasting often seen in these cases.

The answer to the question, What constitutes excess? depends upon the kind, the method of preparation, and the quantity of tea on the one hand, and the idiosyncrasy of the subject towards tea, the state of the digestive and nervous systems, and the occupation of the individual on the other. For anyone who has developed severe symptoms there must be no half-measures: tea must be absolutely withdrawn. It is remarkable what a small allowance of tea will maintain the trouble once severe symptoms have been produced. In milder cases instruction in the art of making tea and strict limitation to a reasonable quantity of a good tea will often do all that is required. Where tea is still allowed very careful and full directions must be given. To tell patients that they must take China instead of Indian tea is as useless as to tell a smoker to give up Virginian cigarettes and take to Turkish. It may be an improvement or it may not. "Four minutes on the leaves" is a rule often given, and religiously applied by the patient—to the first cup. Most people judge of the strength of a cup of tea in the first place by the colour—an unreliable standard that has largely influenced the tea market, and the desire for full value for one's money is not confined to the very poor.

As with alcoholic beverages, the great difficulty is to find a substitute that has both an attractive flavour and a pleasant effect without any reaction from that effect. Coffee is a substitute in little but name, and cocoa is not well borne by those whose digestion has been impaired by tea. The various meat extracts are valuable as temporary substitutes in some cases, but they are expensive and are generally too salty. A German preparation called malt-coffee is useful, but not easy to obtain.

Regulation of the bowels and an alkaline gentian mixture with nux vomica are most efficacious; indeed, in cases where the cause of the trouble has been overlooked they alone may somewhat relieve the symptoms for a time.

The discovery of a history of flagrant excess in tea-drinking must not prevent careful inquiry into other causes of dyspepsia and neurasthenia, such as oral sepsis, the menopause, and worry, since excess in tea-drinking is so often caused in the first instance by ill-health due to some other cause.

For the majority of people tea, properly prepared and taken in moderate quantities, is not only harmless but useful; yet a glance at the pharmacology of two of the active ingredients of tea—caffein and tannin—will show that excess of either may be expected to produce a serious disturbance of health. This disturbance of health, although varying greatly in degree, is so consistent in form that excess in tea-drinking should rarely be overlooked.

